# Prefabricated Bar System for Immediate Loading in Edentulous Patients: A 5-Year Follow-Up Prospective Longitudinal Study

**DOI:** 10.1155/2018/7352125

**Published:** 2018-02-27

**Authors:** Enrico F. Gherlone, Gianpaolo Sannino, Andrea Rapanelli, Roberto Crespi, Giorgio Gastaldi, Paolo Capparé

**Affiliations:** ^1^Department of Dentistry, IRCCS San Raffaele Hospital and Dental School, Vita Salute University, Via Olgettina, No. 48, 20123 Milan, Italy; ^2^San Rocco Clinical Institute, Via dei Sabbioni, No. 24, Ome, 25050 Brescia, Italy

## Abstract

**Objectives:**

The aim of this clinical study was to evaluate a new type of prefabricated bar system, supported by axial and tilted implants at 5-year follow-up.

**Materials and Methods:**

Twenty-nine consecutive participants (19 females, 10 males) (mean age 61.4 years), edentulous in one or both jaws, with severe atrophy of the posterior regions, were treated according to the All-on-four® protocol with immediately loaded axial (64) and tilted (64) implants supporting complete-arch screw-retained prostheses (12 maxillary, 20 mandibular) featuring a prefabricated bar as framework. Follow-up visits were performed at 3, 6, 12, 24, 48, and 60 months after implant insertion. Radiographic assessments were made using panoramic radiographs obtained immediately after surgery and at each follow-up visit. Bone level measurements around the axial and tilted implants were compared by means of the Student's *t*-test.

**Results:**

One axial implant failed in the lower jaw and did not compromise prosthetic function. The 60-month overall implant survival rate was 100% for axially positioned implants and 98.44% for tilted implants. The implant survival rates were 100% in the maxilla and 98.75% in the mandible. None of the 32 fixed prostheses were lost during the observation period, representing a prosthetic survival rate of 100%. No statistically significant differences (*P* > 0.05) in marginal bone loss between tilted and axial implants were detected in either jaw over time.

**Conclusions:**

The use of the evaluated prefabricated bar for immediately loaded implants placed according to the All-on-four concept may significantly reduce implant failures; however, more long-term prospective clinical trials are needed to affirm the effectiveness of the surgical-prosthetic protocol.

## 1. Introduction

Clinical implant dentistry is oriented to low cost treatments using simple protocols that are well supported by scientific data, while providing immediate function through immediate restoration and loading of dental implants [1–4].

The All-on-four concept that employs tilted implants to restore edentulous patients has been proposed as an alternative to bone augmentation procedures [5].

The placement of four implants, two implants tilted posteriorly and two vertical implants in the anterior region, allows for avoiding bone augmentation procedures when rehabilitating a completely edentulous jaw with minimal bone volume [6]. The development to tilting of fewer implants has been encouraged by the results from implant load analyses, demonstrating that four implants are enough for complete-arch prosthesis [7, 8].

Longer implants may be optimally placed in areas with good cortical anchorage to increase prosthetic support and reduce the length of the cantilever. This procedure supported a simpler, less expensive, and less time-consuming treatment compared to maxillary sinus lift or bone grafts [9, 10].

Krekmanov et al. treated forty-seven consecutive patients with implants placed in tilted positions: the cumulative success rates in the maxilla at 5 years were 98% for tilted implants and 93% for nontilted implants [11].

Analysis of the load distribution in one mandibular case showed no significant difference between tilted and the nontilted implants, and the improved prosthesis support was confirmed. The immediate loading of tilted implants with a provisional restoration has been proposed as a simpler, more predictable, less expensive, and less time-consuming treatment of the atrophic maxilla [12]. However a passive fit of the framework plays a key role in splinting and loading nonparallel implants. Tensile, compressive, and bending forces may be dangerous for the osseointegration process and/or result in failure of the components [13].

Soldering or laser welding procedures are often needed to compensate loss of accuracy due to clinical/laboratory errors and achieve the appropriate adaptation of the framework. Using prefabricated parts to assemble prosthetic frameworks, such as bars, could be useful in reducing material distortion, chair time, and the high costs of fabrication. Therefore the aim of this clinical study was to evaluate the use of a prefabricated bar system for immediately loaded implants in patients rehabilitated according to the All-on-four concept with up to 5 years' follow-up.

## 2. Materials and Methods

### 2.1. Patient Selection

This prospective longitudinal study was performed at the Department of Dentistry, San Raffaele Hospital, Milan, Italy. Between March 2011 and March 2012, 29 participants (19 women, 10 men), aged between 41 and 72 years (mean age 61.4) were consecutively treated according to the All-on-four protocol with immediately loaded axial (64) and tilted (64) implants supporting complete-arch screw-retained prostheses (12 maxillary, 20 mandibular). Four patients were treated with both maxilla and mandibular prosthetic rehabilitation.

The investigation was conducted according to the tenets of the Helsinki Declaration and followed STROBE (Strengthening the Reporting of Observational Studies in Epidemiology) guidelines (https://www.strobe-statement.org). The study was approved by the appropriate ethics committees related to the institution in which it was performed and written informed consent was obtained from each participant. The following inclusion criteria were adopted: all patients were in good health, patients had to be edentulous (in one or both jaws) or they had to have a few hopeless teeth, severe atrophy of the mandible or maxilla in posterior regions. Exclusion criteria were the presence of any active infection or severe inflammation in the areas intended for implant placement, presence of chronic systemic disease, any interfering medication such as steroid therapy or bisphosphonate therapy, radiation therapy to head or neck region within 5 years, smoking more than 15 cigarettes, bruxism habits, and poor oral hygiene.

**Figure 1:**
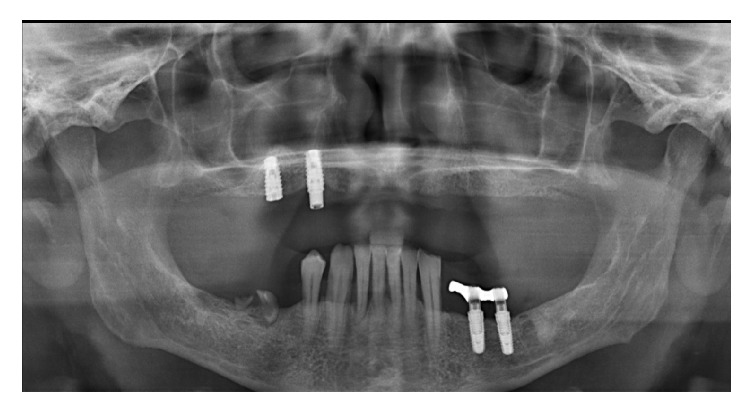
A preoperative panoramic radiograph.

The diagnosis was made clinically and radiographically (preoperative panoramic radiograph and CT scan) (Figure 1). Study casts were obtained from jaw impressions of the patients and mounted on articulators. For edentulous patients, new removable prostheses were fabricated in order to restore optimal occlusal vertical dimension, mandibular position, and occlusal planes. The new prostheses were duplicated and used as radiographic templates during the CT exams. The bone volume was accurately assessed for a safe and prosthetically driven implant placement. All patients gave their written informed consent for immediate implant loading.

### 2.2. Surgical Procedure

One hour prior to surgery the patients received 2 g amoxicillin (Zimox, Pfizer Italia, Latina, Italy) and 1 g twice a day for a week after surgical procedure. Surgery was performed under anesthesia induced by local infiltrations of optocain solution with adrenaline 1 : 80.000 (AstraZeneca, Milan, Italy).

In edentulous mandibles, incisions were made on top of the alveolar crest, from the first molar on one side to the first molar on the contralateral side with bilateral releasing incisions. Subperiosteal dissection on the lingual and vestibular surfaces was carried out and mental foramina were sited. The most posterior implants were placed close to the anterior wall of the mental loop and were tilted distally about 25–30 degrees relative to the occlusal plane. The posterior implants, which were 4.5 mm in diameter and 15 or 13 mm in length, typically emerged at the second premolar position. Anterior implants were either 4.5 or 3.8 mm in diameter and 13 mm in length (Winsix, Biosafin, Ancona, Italy) (Table 1). After placement of the posterior implants bilaterally, additional implants were placed in the anterior space.

In some cases tooth extractions were carried out and, when necessary, bone shaping was performed with a round bur to level the bone crest and, to achieve crestal positioning, bone recontouring was performed distal to the angled implants.

**Figure 2:**
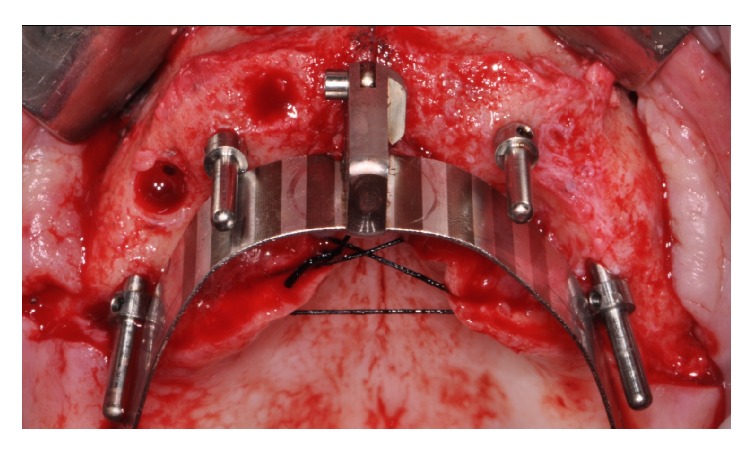
Implant site preparations assisted by a customized surgical guide.

In edentulous maxillary patients, incisions were made on the alveolar crest from the first molar on one side to the first molar on the contralateral side with bilateral releasing incisions. Subperiosteal dissection was carried out. The most posterior implant was placed close to and parallel with the anterior sinus wall. Thus, this implant was tilted distally approximately 25 to 30 degrees. The lower corner of the implant neck was positioned at bone level (Figure 2).

Then the placement of implants in the anterior part of the maxilla was performed, and the implant neck was positioned at bone level. The posterior implants were 4.5 mm in diameter and 15 or 13 mm in length, and the anterior implants were either 4.5 or 3.8 mm in diameter and 13 mm in length (Winsix, Biosafin, Ancona, Italy) (Table 1).

Underpreparation was performed in soft bone to obtain high primary stability. The implant in immediate function had a final insertion torque ranging between 30 and 40 N/cm. Angulated abutments (Extreme Abutment, EA® Winsix, Biosafin, Ancona, Italy) for anterior implants were set at 17° and those for posterior implants at 30° to compensate for the lack of parallelism between implants as well as to place the prosthetic screw-access holes in an occlusal or lingual location. The angulated abutments were tightened with 25 N/cm of torque. Flap adaptation and suturing were performed in the usual manner with 4–0 nonresorbable suture (Vicryl; Ethicon, Johnson & Johnson, New Brunswick, NJ, USA).

Nonsteroidal anti-inflammatory drugs (Brufen 600 mg, Abbott Laboratories, Chicago, IL, USA) and chlorhexidine digluconate 0.2% mouthwash during the first 2 weeks after surgery were prescribed as postoperative care for all participants.

### 2.3. Prosthetic Protocol

**Figure 3:**
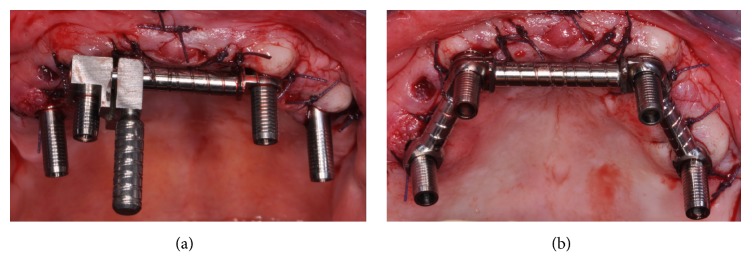
Each tube was shortened at the correct implant distance (a) and connected to adapters by using two dedicated bar joints in order to develop the whole bar structure (b).

**Figure 4:**
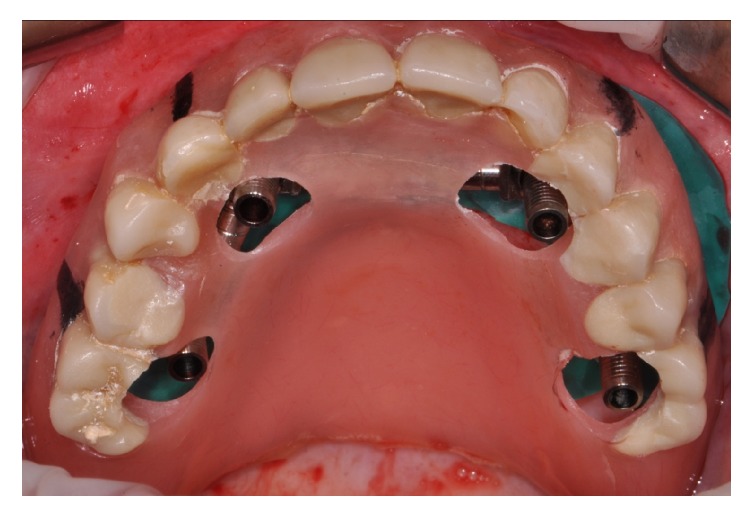
Passive seating and occlusal relationship of the removable prostheses were checked. Removable prostheses were released in correspondence with the expected bar volume. The contacts between the prostheses and the mucosal regions, which were not involved in the surgical procedures, were used as anatomical pre- and postsurgical landmarks to avoid variations in the occlusal relationships previously achieved. Four large openings were made according to planned cylinder emergence.

After surgery straight cylinders (AT, WINSIX; BiosafIn) were screwed onto the angulated abutment. The interimplant distance was measured (Figure 3(a)). Three bar tubes were shortened to the optimal lengths using the specific cutter bar device and a splitter disk [14]. Two bar joints (CF and CM, WINSIX; BiosafIn) were inserted into the end of each tube in order to connect the whole structure to the cylinders (Figure 3(b)). The height of the cylinders was chosen to make the bar parallel to the occlusal plane. All the joints were connected to the cylinders and fixed by means of resin cements. No soldering was performed; the universal nature of the ball joint allows the tube bar to be located in the horizontal plane in a truly stress free alignment [15]. The prostheses were provided with four large openings according to planned cylinder emergence. The passive seating and the occlusal relationship of the removable prostheses were checked (Figure 4). The bar system was attached to the denture with self-curing acrylic resin.

**Figure 5:**
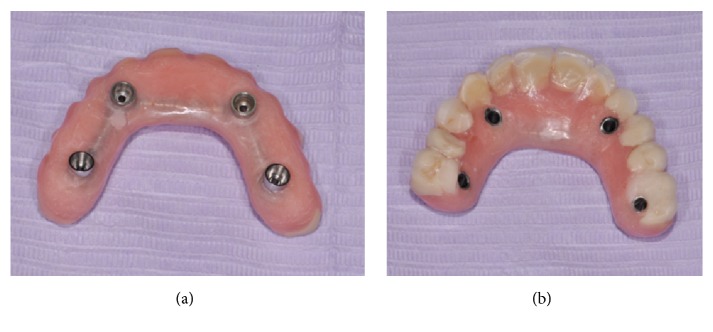
The bar was attached to the denture with self-curing acrylic resin and marginal precision was improved by resin addition around the collar of the adapters ((a) crestal view, (b) occlusal view).

**Figure 6:**
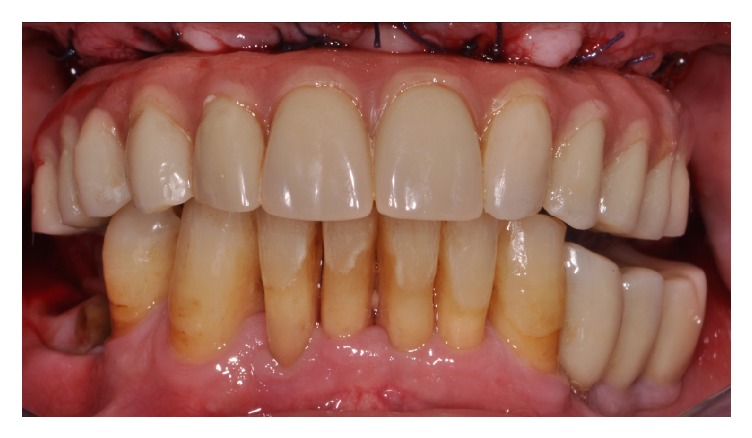
Provisional screw-retained bar-reinforced acrylic resin prosthesis delivery. Frontal view.

After polymerization, the prostheses with the incorporated bar system were removed from the implants and retention, marginal precision, and stability were improved by resin addition around the collar of the cylinders (Figures 5(a) and 5(b)). Screw-retained full-arch temporary prostheses were placed (Figure 6).

Cantilevers were extended to the first molar regions and in three cases only to second premolar. Articulating paper (Bausch Articulating Paper, Nashua, NH, USA) was used to check the occlusion and adjust it, if necessary. Static occlusion consisted of central contacts established on all masticatory units. Dynamic occlusion included canine/premolar guidance, regardless of the opposite arch settings. Screw-access holes were covered with provisional resin (Fermit, Ivoclar Vivadent, Naturno, Bolzano, Italy). Fifteen days after prosthesis delivery, a final occlusal adjustment was performed.

All patients followed a soft/liquid diet for 2 months (the bread consistency varied).

**Figure 7:**
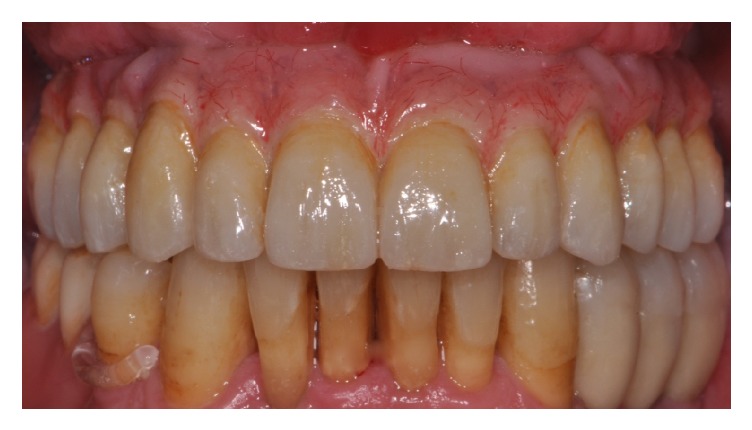
Definitive screw-retained full-arch restoration at 60-month follow-up.

After 4 months from implant positioning, 32 definitive prostheses were placed (Figure 7).

### 2.4. Follow-Up

Follow-up visits were performed by a dental hygienist, trained for clinical studies, and calibrated at 3, 6, 12, 24, 36, 48, and 60 months after implant insertion. Success criteria for implant survival were presence of implant stability, absence of radiolucent zone around the implants, no mucosal suppuration, and no pain. Restoration success was defined as the absence of fractures of the acrylic resin superstructure. Implant survival was defined as the absence of implant mobility, swelling, or pain in the surgical site at the time of examination.

Implant success was defined as implant survival plus marginal bone loss of less than 1.5 mm after 1 year of loading and no more than 0.2 mm of loss between each follow-up appointment after the first year of function.

### 2.5. Radiographic Examination

**Figure 8:**
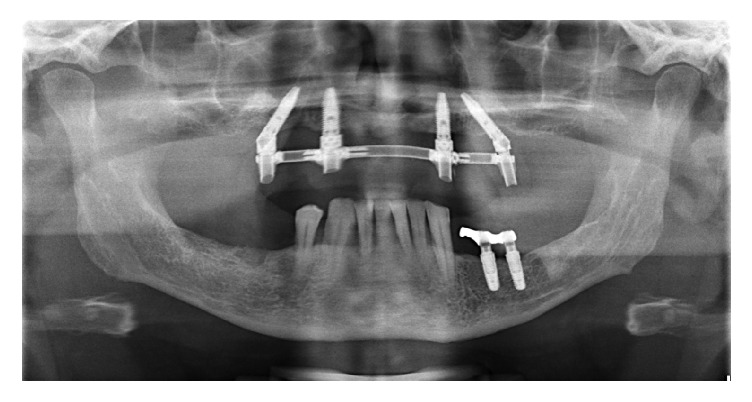
Panoramic radiograph at 60-month follow-up.

Radiographic assessments were made using panoramic radiographs obtained immediately after surgery and at each follow-up visit (Figure 8). Bone level measurements were performed on the mesial and distal aspect of each implant, using the implant-abutment junction as a reference point.

To adjust for dimensional distortion and enlargement on the radiographs, the actual sizes of the implants were compared to the measured implant dimensions on the radiograph [1, 15]. A radiologist twice measured the changes in marginal bone height over time: the reference points were marked and the lines were measured on the screen interactively (the numeric value of measurements was reported by software) (CDR, Schick Technologies, Long Island City, NY, USA). The implant length (a known dimension) was used for calibration. The radiographic measurements were compared to the values obtained immediately after surgery.

### 2.6. Statistical Analysis

A dedicated software (SPSS 11.5.0, SPSS, Chicago, IL, USA) was used for all statistical analyses. Sample size calculation was performed before patients recruitment. Bone level measurements were reported as means ± standard deviations at 6, 12, and 60 months. Bone loss around the upright and tilted implants was compared by means of the Student's *t*-test at a significance level of *P* = 0.05.

## 3. Results

In the first four months after implant placement, one implant failed (one mandibular), as a result of painfulness (Table 2). The failed implant was axial and did not compromise prosthetic function. An implant of the same length and larger diameter was immediately placed and left unloaded until a new definitive prosthesis was completed and inserted.

The 5-year overall implant survival rate was 100% for axially positioned implants and 98.44% for tilted implants. The implant survival rates were 100% in the maxilla and 98.75% in the mandible.

None of the 32 fixed prostheses were lost during the observation period, representing a prosthetic survival rate of 100%. Occlusal screw loosening, was observed in 3.03% of cases (4 implants) within 6 months of follow-up.

Radiographic results are reported in Table 3. At the 60-month evaluation, peri-implant crestal bone loss averaged 1.08 ± 0.45 mm for upright maxillary implants (*n* = 24 implants) and 1.02 ± 0.67 mm for tilted maxillary implants (*n* = 24 implants) (Table 3). In the mandible, a mean peri-implant crestal bone loss of 1.04 ± 0.61 mm for upright implants (*n* = 40) and 1.09 ± 0.56 mm for tilted implants (*n* = 40) was found (Table 3).

No statistically significant differences (*P* > 0.05) in crestal bone loss between tilted and upright implants were detected at 6-, 12-, and 60-month follow-up evaluation in either jaw.

## 4. Discussion

The data from the present prospective study have shown encouraging clinical results as a means of restoring edentulous jaws with immediately loaded full-arch fixed prostheses supported by a prefabricated bar system and screwed onto two anterior axial implants and two distal tilted implants.

One loaded implant was lost, and the prosthesis survived on the remaining three implants until the replacement implant was loaded. The use of three loaded implants allows for the failure of one implant without failure of the prosthesis. The failed implant was inserted with a torque of at least 40 Ncm.

In the current study, radiographs demonstrated that the bone resorption pattern for posterior, angulated implants was similar on the mesial and distal surfaces. This was in agreement with the findings of other studies [8–10, 16–18].

No statistically significant differences (*P* > 0.05) in crestal bone loss between tilted and upright implants were detected at 6- and 12-month follow-up evaluation in either jaw, and this is also consistent with other data found in the literature, confirming that tilted implants may achieve the same outcome as implants placed in an upright position [6, 16]. This positive result is associated with biomechanical advantages, since in this protocol implants are placed in strategic positions from a load-sharing point of view. Placement of the 2 well-anchored posterior tilted implants together with the anterior upright implants can provide a predictable foundation for an implant-supported prosthesis. This surgical-prosthetic procedure also seems to validate the reduced length of the prostheses cantilevered segments. Implant placement and orientation provided effective cross-arch stabilization without the need for bone grafting procedures. Excluding maxillary sinus bone grafts resulted in significantly less morbidity and dramatically decreased the financial costs associated with those procedures.

This treatment protocol allows the implant rehabilitation to be simplified and shortened for both the patient and the clinical team. The postsurgical period is more comfortable for patients since they have been utilizing their fixed prosthesis from the first day [9].

The immediate creation of the temporary restoration with a simple and repeatable prosthetic protocol represents a major advantage for patients, providing less expensive and less time-consuming treatments [10].

Traditional laboratory procedures such as soldering and welding may give rise to errors and increase in cost; furthermore, several bar framework material, such as gold alloy, silver-palladium alloy, commercially pure titanium, and cobalt-chromium alloy transfer significant stress to the supporting peri-implant tissues [19].

The key of prefabricated precision-milled components is that the framework is assembled without the use of soldering, laser welding, or conventional bonding techniques, thus reducing stress transmission to bone around the implants.

There is no casting, soldering, laser welding, or bonding of components when fabricating the definitive bar. This, combined with the universal ball joint nature of the components, ensures a true passive fit when the bar is assembled and seated.

No laboratory time is required to fabricate the bar and there are no gold-alloy charges. Clinically, there is no need for the bar sections to be soldered in an attempt to achieve passive fit—a step that may need repeating—as with the conventional method. The prefabricated bar is relatively inexpensive compared with conventional gold castings and CAD/CAM options.

Precision-milled components provide an improved quality of fit. The physical and mechanical properties of the component materials can be controlled accurately, which is difficult to achieve with conventional casting methods [14]. The passive-fit bar assembly can result in greatly reduced stress transmission to the supporting implants [14]. Studies have demonstrated that this is also a viable treatment option for immediate-loading situations in the mandible, provided that the implants achieved insertion torques exceeding 50 Ncm approximately [20].

A clinical study [21] evaluated initial, 4-month, and 1-year stability of immediately loaded dental implants inserted according to a protocol of lower rehabilitation with prefabricated bars. Immediately after implant installation, resonance frequency analysis (RFA) for each implant was registered as well as after 4 months and 1 year with the prosthetic bar removed as it is a screwed system. The analysis of variance showed a statistically significant result (*P* = 0.015) among implant stability quotient values for the different periods evaluated. Tukey test results showed statistically significant differences between 1-year results and the initial periods but there was no statistically significant difference between initial and 4-month results (*P* > 0.05).

Prefabricated bars were compared to custom-made bars used for implant-retained mandibular complete overdentures [22]. All patients were evaluated clinically and radiographically immediately after overdenture delivery and after 6, 12, and 18 months.

There was more pronounced bone resorption in cast bar group more than the prefabricated bar group and minimal marginal bone loss in the group treated with prefabricated bar. The prefabricated bar overdentures showed less bone resorption distal to the implants in comparison with the cast bar implant-retained overdentures. The prefabricated bar implant-retained overdenture showed low significant reduction in the bone height after 1 year, and a very highly significant reduction after 18 months.

## 5. Conclusions

Data and results of this clinical study demonstrated high success rates and a low number of complications.

The use of the evaluated prefabricated bar for immediately loaded implants placed according to the All-on-four concept may significantly reduce implant failures; however, more long-term prospective clinical trials are needed to affirm the effectiveness of the surgical-prosthetic protocol especially performed in various clinical centers from different clinicians.

## Figures and Tables

**Table 1:** Implant diameters and lengths for maxilla and mandible (maxilla *n* = implant = 48; mandible *n* = implant = 80).

		Length 13 mm	Length 15 mm
Maxilla *n* = 48			
Upright *n* = 24	Diameter 4.5 mm	10	0
	Diameter 3.8 mm	14	0
Tilted *n* = 24	Diameter 4.5 mm	12	12
	Diameter 3.8 mm	0	0

Mandible *n* = 80			
Upright *n* = 40	Diameter 4.5 mm	4	10
	Diameter 3.8 mm	8	18
Tilted *n* = 40	Diameter 4.5 mm	18	14
	Diameter 3.8 mm	4	4

**Table 2:** Failure table for upright and tilted implants (maxilla *n* = implant = 48; mandible *n* = implant = 80).

	Placed	Failed	Survival (%)
Maxilla *n* = 48			
Upright	24	0	100
Tilted	24	0	100

Mandible *n* = 80			
Upright	40	1	97.50
Tilted	40	0	100

**Table 3:** Crestal bone loss values (mean ± SD) for maxillary and mandibular tilted and upright implants (maxilla *n* = implant = 48; mandible *n* = implant = 80).

Bone Loss	Upright		Tilted	
	Maxilla *n* = 24	Mandible *n* = 40	Maxilla *n* = 24	Mandible *n* = 40
6 months (mm)	0.99 ± 0.23	0.97 ± 0.31	1.00 ± 0.32	1.01 ± 0.27
12 months (mm)	1.03 ± 0.33	1.05 ± 0.44	1.06 ± 0.50	1.08 ± 0.41
60 months (mm)	1.08 ± 0.45	1.04 ± 0.61	1.02 ± 0.67	1.09 ± 0.56
